# Prostacyclin and PPARα Agonists Control Vascular Smooth Muscle Cell Apoptosis and Phenotypic Switch through Distinct 14-3-3 Isoforms

**DOI:** 10.1371/journal.pone.0069702

**Published:** 2013-07-03

**Authors:** Yen-Chung Chen, Ling-Yun Chu, Shu-Fan Yang, Hua-Ling Chen, Shaw-Fang Yet, Kenneth K. Wu

**Affiliations:** 1 Institute of Cellular and System Medicine, National Health Research Institutes, Zhunan, Miaoli, Taiwan; 2 Institute of Biotechnology, College of Life Science, National Tsing Hua University, Hsin-Chu, Taiwan; 3 Metabolomic Medicine Research Center, China Medical University, Taichung, Taiwan; Universitat de Lleida - IRBLLEIDA, Spain

**Keywords:** prostacyclin, peroxisome proliferator-activated receptor, smooth muscle cell, 14-3-3, apoptosis, phenotypic switch

## Abstract

We hypothesized that prostacyclin (PGI_2_) protects vascular smooth muscle cell (VSMC) against apoptosis and phenotypic switch through peroxisome proliferator-activated receptor-α (PPARα) activation and 14-3-3 upregulation. Here we showed that transfection of rat aortic VSMC, A-10, with PGI_2_-producing vectors, Ad-COPI, resulted in attenuated H_2_O_2_-induced apoptosis accompanied by a selective increase in 14-3-3β and 14-3-3θ expression. Carbaprostacyclin (cPGI_2_) and Wy14,643 exerted a similar effect. The effects of PGI_2_ were abrogated by MK886, a PPARα antagonist, but not GSK3787, a PPARδ antagonist. PPARα transfection upregulated 14-3-3β and θ expression and attenuated H_2_O_2_-induced apoptosis. H_2_O_2_-induced 14-3-3β but not 14-3-3θ degradation was blocked by a caspase 3 inhibitor. Furthermore, 14-3-3β but not 14-3-3θ overexpression reduced, while 14-3-3β siRNA aggravated apoptosis. VSMC contractile proteins and serum response factor (SRF) were reduced in H_2_O_2_-treated A-10 cells which were concurrently prevented by caspase 3 inhibitor. By contrast, PGI_2_ prevented H_2_O_2_-induced SM22α and Calponin-1 degradation without influencing SRF. cPGI_2_ and Wy14,643 also effectively blocked VSMC phenotypic switch induced by growth factors (GFs). GFs suppressed 14-3-3β, θ, ε and η isoforms and cPGI_2_ prevented the decline of β, θ and η, but not ε. 14-3-3θ siRNA abrogated the protective effect of cPGI_2_ on SM22α and Calponin-1 while 14-3-3 θ or 14-3-3β overexpression partially restored SM22α. These results indicated that PGI_2_ protects VSMCs via PPARα by upregulating 14-3-3β and 14-3-3θ. 14-3-3β upregulation confers resistance to apoptosis whereas 14-3-3θ and β upregulation protects SM22α and Calponin-1 from degradation.

## Introduction

Prostacyclin (PGI_2_) is a key mediator of vascular homeostasis [1]. It inhibits platelet aggregation and thereby controls vascular thrombosis. It acts on vascular smooth muscle cells (VSMCs) to regulate vascular tone. Its control of platelet aggregation and VSMC contraction is mediated via the plasma membrane I-type prostanoid (IP) receptors [2]. PGI_2_ was subsequently reported to possess other biological activities such as apoptosis control [3,4] and embryo development and implantation [5], which are mediated via peroxisome proliferator-activated receptors (PPARs) [6,7]. Stable analogs of PGI_2_ bind and activate PPARα and PPARδ [8,9]. PPARs are nuclear receptors which in cooperation with retinoid X receptors transactivate diverse effector genes [10]. 14-3-3 comprises seven isoforms in mammals which function as scaffolds to integrate the actions of diverse proteins including kinases, transcription factors, apoptotic molecules [11,12]. We discovered that PGI_2_ and its stable analog, carbaprostacyclin (cPGI_2_) protect vascular endothelial cell (VEC) from oxidant-induced apoptosis by upregulating the 14-3-3ε isoform which enhances Bad sequestration and attenuates Bad-induced apoptosis [13]. It is unclear whether PGI_2_ protects vascular smooth muscle cell (VSMC) through 14-3-3 upregulation.

Vascular endothelial cells produce PGI_2_ and release it into blood and the vascular wall where it controls blood platelet activation and protects VECs and VSMCs. Under normal condition, PGI_2_ production in VEC is stimulated by shear stress [14]. When VECs encounter stress signals from endotoxins, cytokines, environmental toxins and immune mediators, they express abundant COX-2 to defend against the insults [15]. It was recently reported that PGI_2_ generated from VECs controls VSMC phenotypic switch via PPAR [16], suggesting that PGI_2_ exerts a broad influence on VSMC function. VSMCs normally reside in the medial layer of blood vessels, and assume a quiescent state. They express VSMC-specific contractile proteins to confer smooth muscle contractility [17]. Upon vascular injury and platelet activation, VSMCs migrate to the intimal layer and undergo phenotypic switch: they lose contractile proteins and gain proliferative and synthetic functions. That VEC-produced PGI_2_ is capable of preventing phenotypic switch underscores the importance of vascular auto-protection conferred by PGI_2_. However, it is unclear how PGI_2_ preserves VSMC contractile proteins. We hypothesized that PGI_2_ prevents VSMC from apoptosis and contractile phenotypic switch by related common mechanisms. The results provide evidence to support this. Our data show that PGI_2_ and PPARα agonists protect VSMC from H_2_O_2_-induced caspase 3 activation and apoptosis through PPARα-mediated 14-3-3β upregulation, and preserve SM22α and Calponin-1 via 14-3-3θ and β upregulation.

## Materials and Methods

### Reagents and antibodies

Carbaprostacyclin (cPGI_2_), Wy14,643, GW9578 and MK886 were purchased from Cayman Chemical. GSK3787 was purchased from TOCRIS. Z-DEVD-fmk was purchased from Biovision. H_2_O_2_ and ABT-737 were purchased from Calbiochem (Merck Chemicals). Mouse monoclonal antibody against 14-3-3β, rabbit polyclonal antibodies against 14-3-3 isoforms (ε, γ, ξ, and θ) and PGI_2_ synthase (PGIS), goat monoclonal antibodies against 14-3-3η and HSP60 were purchased from Santa Cruz Biotechnology. Rabbit polyclonal antibodies against cleaved caspase 3, cleaved poly(ADP-ribose) polymerase (PARP), Bad and serum response factor (SRF) were purchased from Cell Signaling Technology. Monoclonal antibody against Flag, β-actin and SMA were purchased from Sigma-Aldrich. Rabbit polyclonal antibody against SM22α was purchased from ***Abcam***. Rabbit monoclonal antibody against calponin-1(CPN) was purchased from Millipore. Platelet-derived growth factor-BB (PDGF-BB) was purchased from Sigma-Aldrich. Fibroblast growth factor-basic (FGF2) was purchased from PeproTech. Epidermal growth factor (EGF) was purchased from PROSPEC Protein Specialists.

### Cell Culture and treatment

Rat thoracic aorta smooth muscle cells, A-10, were purchased from Bioresource Collection and Research Center (BCRC) and cultured in Dulbecco’s modified Eagle’s medium (DMEM) (***GIBCO***) supplemented with 10% fetal bovine serum (FBS) (Hyclone), 100 U/ml penicillin, and 100 µg/ml streptomycin (***GIBCO***) at 37°C in a humidified 5% CO_2_ atmosphere. In initial experiments, A-10 cells were treated with various concentrations of H_2_O_2_ for periods of time and apoptosis was determined. We found treatment of A-10 with 0.8 mM H_2_O_2_ for 6 h (Figure S1) to be optimal. To evaluate the effects of cPGI_2_, MK886 (a PPARα antagonist, 25 µM), GSK3787 (a PPARδ antagonist, 25 µM), or ABT-737 (a Bcl-2 inhibitor, 1 µM) on apoptosis, A-10 cells were pretreated with the pharmacological compound for 2 h before treatment with H_2_O_2_ for 6 h. For VSMC phenotype switch experiments, A-10 cells were pretreated with cPGI_2_ or Z-DEVD-fmk (20 µM) for 2 h before treatment with H_2_O_2_ for 6 h or growth factors (PDGF; 20 ng/ml, FGF2; 2 ng/ml and EGF; 0.5 ng/ml) for 48 h.

### Plasmid construct, siRNA and transfection

cDNA of PPARα was amplified by PCR and cloned into the p3XFlag-CMV expression vector (Sigma-Aldrich) with the restriction enzymes ClaI and XbaI. p3XFlag-14-3-3 isoform plasmids were kindly provided by Dr. Jun-Yang Liou at NHRI, Taiwan. DNA plasmids were transfected into A-10 cells using GenJet (SignaGen Laboratories) for 48 h before treatment with H_2_O_2_ for 6 h. For siRNA transfection, A-10 cells were transfected with the designated siRNA or scramble siRNA (scRNA) (Santa Cruz Biotechnology) for 48 h using GenMute (SignaGen Laboratories).

### Western blot analysis

50 µg of cell lysate proteins were loaded to 12% SDS-PAGE gels and transferred to nitrocellulose membranes (Bio-Rad). The membranes were then blocked with 5% non-fat milk and incubated with specific primary antibodies overnight at 4°C, washed and incubated with HRP-conjugated secondary antibodies for 1 h at room temperature. Signals were revealed using an ECL chemiluminescence Kit (Thermo Scientific). Blots were quantified by scanning and analyzed by ImageJ software (National Institute of Health).

### Immunofluorescence staining

A-10 cells were fixed with 4% paraformaldehyde for 15 min, washed, and after treatment with 5% goat normal serum-0.3% Triton x-100-PBS for 1 h, they were incubated overnight at 4°C with cleaved caspase-3 antibody or SM22α antibody in 1% BSA-PBS followed by incubation with FITC-conjugated secondary antibody. The fluorescent image was detected with Leica DM2500 Upright Fluorescence Microscope.

### Recombinant adenoviral vectors

The PGI_2_-producing adenoviral vector, Ad-COPI, contains a bicistronic cyclooxygenase-1 (COX-1) and PGI_2_ synthase (PGIS) construct which induces COX-1 and PGIS overexpression resulting in robust PGI_2_ production [18]. It was generated by homologous recombination and amplified in 293 cells as described previously [18]. A-10 cells were infected with recombinant adenovirus for 48 h before treatment with H_2_O_2_ for 6 h. An empty adenovirus (Ad-null) was used as a control.

### Preparation of mitochondrial fraction

Mitochondrial fractions were prepared using a mitochondria isolation kit from Thermo Scientific. The mitochondrial pellets were lysed in RIPA lysis buffer (Millipore) and stored at -20°C. Heat shock protein 60 (HSP60) was used as a mitochondria marker.

### Immunoprecipitation

cPGI_2_-or Wy14,643-treated A-10 cells were harvested and immunoprecipitated with a Bad antibody. The immunoprecipitated complex was pulled down with protein A magnetic beads (Millipore). After washing 5 times, the proteins were analyzed by Western blotting using Bad and 14-3-3β antibodies.

### Statistical Analysis

Values are expressed as mean±SEM as indicated in the figure legends. Differences between groups were analyzed using One Way ANOVA with SigmaStat software (Systat Software, Inc.). *P*<0.05 was considered statistically significant.

## Results

### Prostacyclin prevents VSMC apoptosis via PPARα

**Figure 1:**
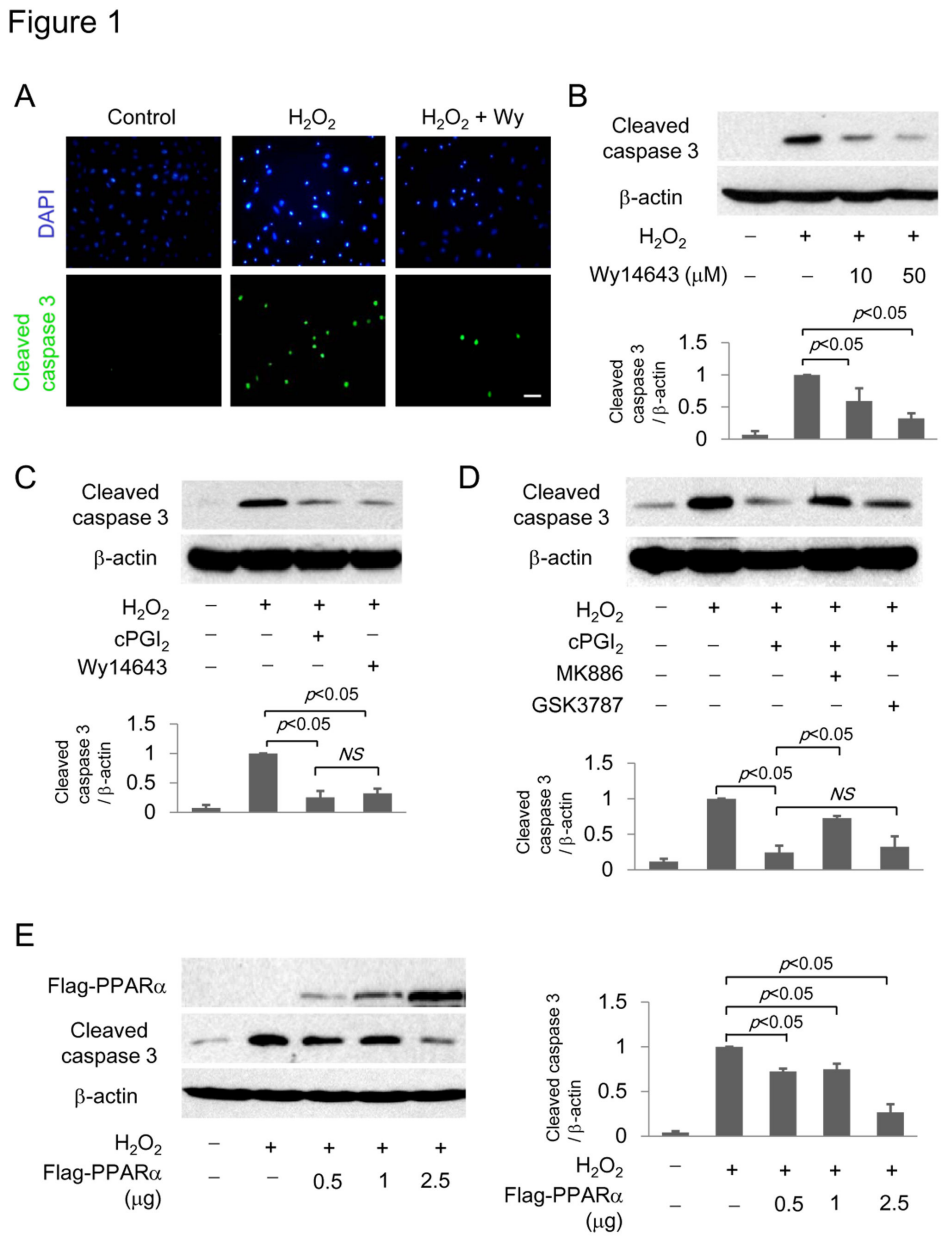
PPARα ligands attenuate H_2_O_2_-induced VSMC apoptosis. (A–C) A-10 VSMCs were pretreated with Wy14,643 (50 µM or otherwise indicated), or cPGI_2_ (100 µM) followed by H_2_O_2_ (0.8 mM). (**A**) Cells were stained with DAPI or immune-stained for cleaved caspase 3 and examined under fluorescent microscope. Scale bar = 100 µm. (**B** and **C**) Cleaved caspase 3 was analyzed by Western blotting. Upper panel shows a representative blot and the lower panel shows the quantitative analysis of densitometry of the Western Blots. (**D**) A-10 cells were pretreated with MK886 (25 µM) or GSK3787 (25 µM) followed by cPGI_2_ (100 µM) and H_2_O_2_ (0.8 mM). Upper panel shows a representative blot and the lower panel shows the quantitative analysis. (**E**) A-10 cells were transfected with Flag-tagged PPARα vectors at different concentrations. PPARα expression was analyzed using a Flag antibody. Cleaved caspase 3 was analyzed by Western blotting using a specific antibody. The left panel shows a representative Western blot and the right panel shows the quantitative analysis. Each error bar denotes mean±SEM and all blots are representative of n≥3. NS denotes statistically non-significant.

We used H_2_O_2_ injury as a model to investigate VSMC apoptosis. H_2_O_2_ at 0.8 mM induced caspase 3 activation as manifested by PARP and procaspase 3 cleavage on Western blot analysis (Figure S1). It caused VSMC nuclear condensation as well as cleaved caspase 3 as analyzed by immunofluorescence microscopy (Figure 1A). Wy14,643, a PPARα agonist at 50 µM, prevented H_2_O_2_-induced nuclear condensation and cleaved caspase 3 as analyzed by immunofluorescence (Figure 1A) and Western blotting in a concentration-dependent manner (Figure 1B). cPGI_2_, a stable PGI_2_ analog at 100 µM, blocked cleaved caspase 3 to an extent comparable to Wy14,643 at 50 µM (Figure 1C). VSMC cultured in serum-free medium for 48 h exhibited PARP cleavage which was inhibited by cPGI_2_ and another PPARα agonist, GW9578 (5 µM) (Figure S2). To ensure that PPARα protects against apoptosis, we pretreated cells with MK886 (25 µM), a PPARα antagonist or GSK3787 (25 µM), a PPARδ antagonist, and analyzed cleaved caspase 3 in cells treated with H_2_O_2_ in the presence or absence of cPGI_2_. MK886 abrogated the protective effect of cPGI_2_ whereas GSK3787 did not significantly influence the anti-apoptotic action of cPGI_2_ (Figure 1D). Conversely, PPARα overexpression by transfection of flag-tagged PPARα vector suppressed cleaved caspase 3 in a dose-dependent manner (Figure 1E). Taken together, these results indicate that PPARα represents a major transcriptional pathway in control of apoptosis and cPGI_2_ prevents VSMC apoptosis primarily via PPARα.

### PGI_2_ and Wy14,643 prevent H_2_O_2_-induced apoptosis by upregulating 14-3-3β

**Figure 2:**
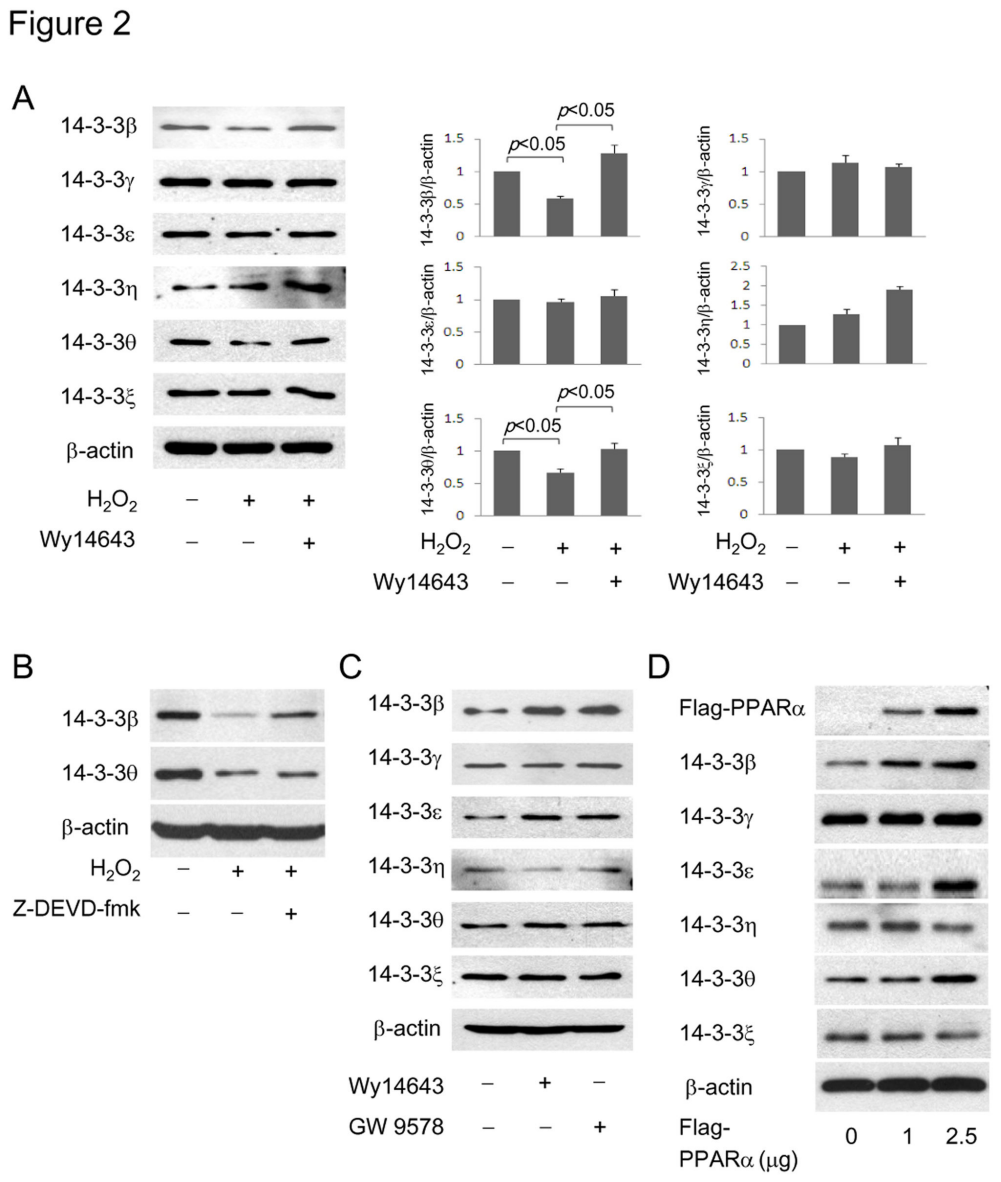
PPARα rescues H_2_O_2_-induced depression of 14-3-3β and θ levels. (**A**) A-10 cells were pretreated with Wy14,643 (50 µM) followed by H_2_O_2_ (0.8 mM). 14-3-3 proteins were analyzed by Western blotting. Left panel shows representative Western blots and right panel shows densitometry analysis. (**B**) Cells were pretreated with caspase 3 inhibitor, Z-DEVD-fmk (20 µM) followed by H_2_O_2_. 14-3-3β and θ were analyzed by Western blotting. (**C**) Cells were treated with Wy14,643 (50 µM) or GW9578 (5 µM) and changes in 14-3-3 proteins were analyzed. (**D**) Cells were transfected with PPARα vectors and 14-3-3 proteins were analyzed. Each error bar denotes mean±SEM and all blots are representative of n≥3.

**Figure 3:**
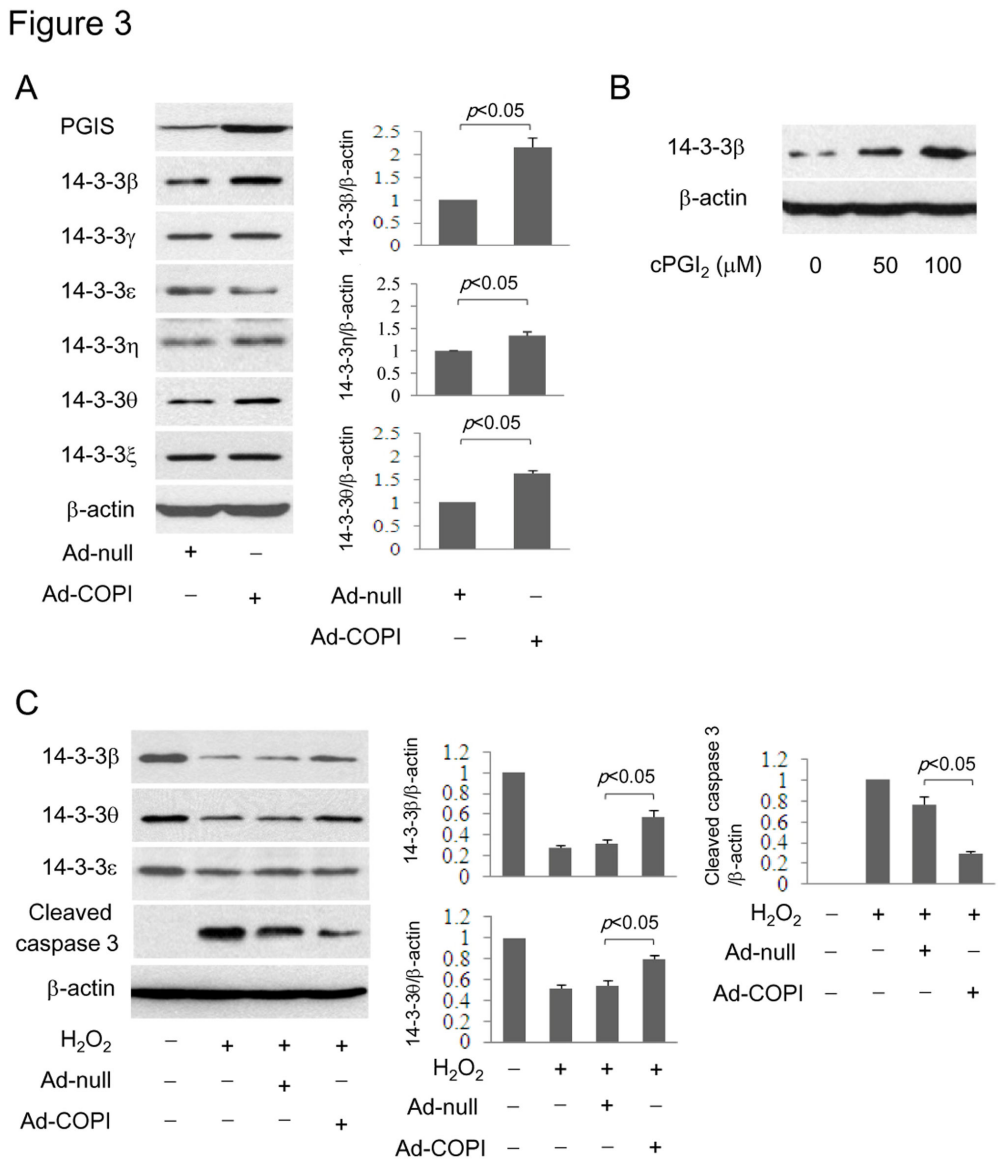
PGI_2_ increases 14-3-3β expression. (**A**) A-10 cells were transfected with Ad-COPI or control adenoviral vector Ad-null. 14-3-3 proteins were determined by Western blotting. Left panel shows representative Western blots and right panel shows densitometry analysis. (**B**) Cells were treated with cPGI_2_. 14-3-3β was measured by Western blotting. (**C**) Ad-COPI or Ad-null transfected cells were treated with H_2_O_2_. 14-3-3 isoforms and cleaved caspase 3 were analyzed. Left panel shows representative Western blots and right panel shows densitometry analysis. Each error bar denotes mean±SEM and all blots are representative of n≥3.

PGI_2_ has been shown to protect vascular endothelial cells from oxidant-induced apoptosis by upregulating the 14-3-3ε [13]. We postulated that PGI_2_ protects VSMC against apoptosis also through 14-3-3 upregulation. To test this hypothesis, we initially determined the 14-3-3 isoforms that might be upregulated by PGI_2_ and PPARα agonists. All seven isoforms except 14-3-3σ were detected in resting VSMCs (Figure 2A). The protein level of 14-3-3β and θ was depressed in H_2_O_2_-treated cells while the level of 14-3-3ε was unaffected (Figure 2A). To determine whether depression of 14-3-3β and θ may be due to caspase-induced protein degradation, we evaluated the effect of Z-DEVD-fmk, a caspase 3 inhibitor, on H_2_O_2_-induced reduction of these two isoforms. Z-DEVD-fmk at 20 µM prevented 14-3-3β but not 14-3-3θ depression by H_2_O_2_ (Figure 2B). The result is consistent with a previous report that caspase 3 degrades 14-3-3β but not θ [19]. H_2_O_2_-induced 14-3-3β and θ reduction was reversed by pretreatment with Wy14,643 (Figure 2A). Wy14,643 (50 µM) and GW9578 (5 µM) increased predominantly 14-3-3β and 14-3-3ε in cells without H_2_O_2_ treatment (Figure 2C). 14-3-3β level was raised in cells transfected with 1 µg PPARα vectors (Figure 2D), and besides 14-3-3β, the θ and ε isoforms were also raised by transfection with 2.5 µg PPARα vectors (Figure 2D). 14-3-3β, θ and η were increased in cells infected with PGI_2_-producing vector, Ad-COPI (Figure 3A). Furthermore, cPGI_2_ increased 14-3-3β in a concentration-dependent manner (Figure 3B). H_2_O_2_-induced 14-3-3β or θ depression was attenuated in Ad-COPI infected cells, which was correlated with reduction of cleaved caspase3 (Figure 3C). It was reported that PPARγ activation increases the expression of anti-apoptotic Bcl-2 family proteins, which contribute to defense against apoptosis [20,21]. We determined whether Bcl-2 is implicated in the anti-apoptotic action of cPGI_2_ by using ABT-737 which inhibits the anti-apoptotic family proteins Bcl-2, Bcl-XL and Bcl-w [22]. We pretreated cells with ABT-737 (1 µM) followed by cPGI_2_ (100 µM) and H_2_O_2_ (0.8 mM). ABT-737 did not influence the anti-apoptotic action of cPGI_2_ (Figure S3). Taken together, these results suggest that PGI_2_ and PPARα agonists upregulate 14-3-3β and θ via which they defend against VSMC apoptosis.

### 14-3-3β controls VSMC apoptosis by binding and sequestering Bad

**Figure 4:**
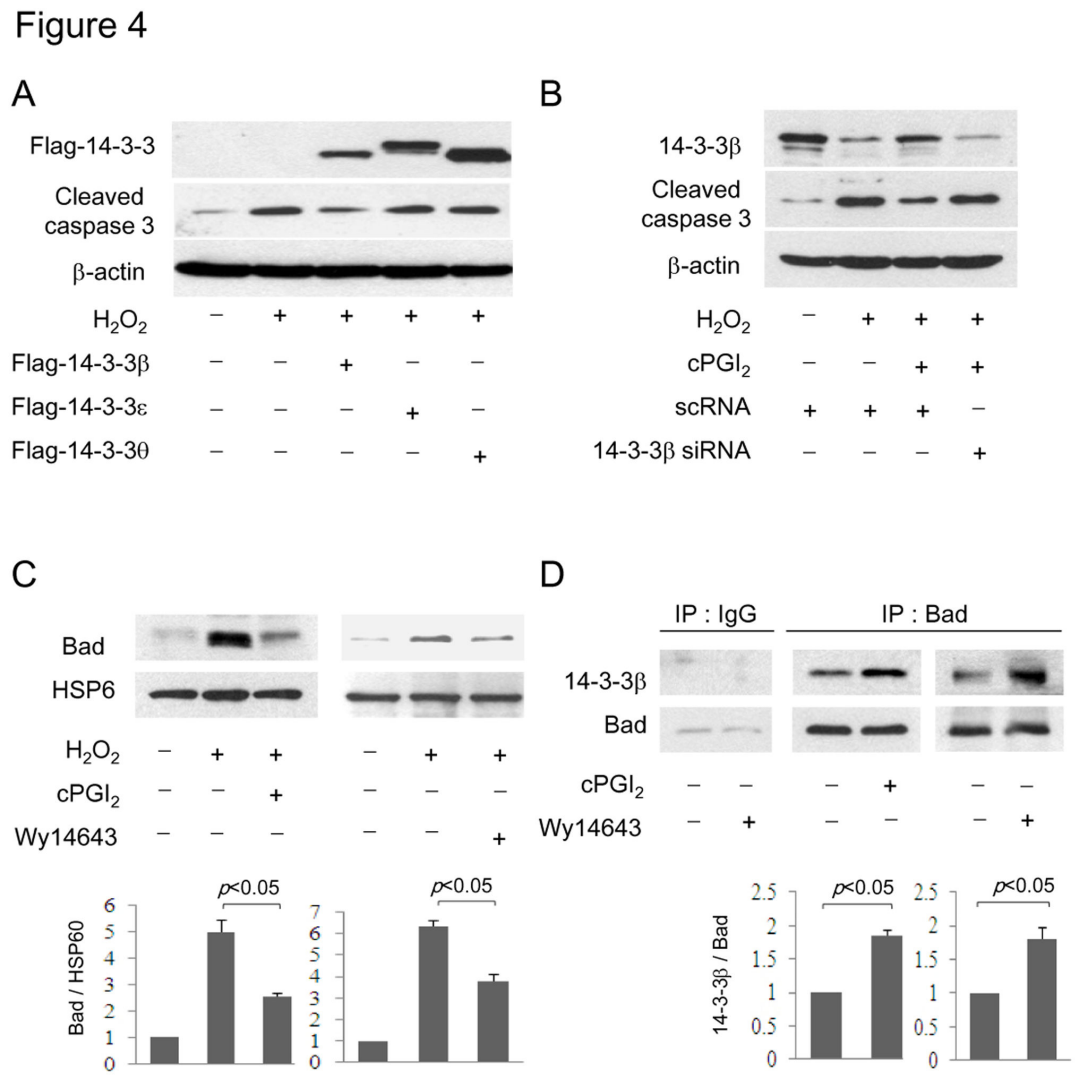
14-3-3β protects against H_2_O_2_-induced apoptosis by sequestering Bad. (**A**) A-10 cells were transfected with Flag-tagged 14-3-3β, ε or θ vectors. Following H_2_O_2_ treatment, cells were lysed and cleaved caspase 3 was determined. (**B**) Cells were transfected with 14-3-3β siRNA or control scRNA. The transfected cells were treated with cPGI_2_ and H_2_O_2_. 14-3-3β and cleaved caspase 3 were analyzed by Western blotting. (**C**) Cells were treated with cPGI_2_ (100 µM) or Wy14,643 (50 µM) prior to H_2_O_2_ treatment. Mitochondrial fractions of VSMCs were isolated and Bad was analyzed by Western blotting. Heat shock protein 60 (HSP60) was concurrently measured as mitochondrial marker. (**D**) Cells were treated with cPGI_2_ (100 µM) or Wy14,643 (50 µM) and then lysed. Lysates were immunoprecipitated with Bad antibody or control IgG. 14-3-3β and Bad in the immunoprecipitates were determined by Western blotting. Each error bar denotes mean±SEM and all blots are representative of n≥3. NS denotes statistically non-significant.

To test the hypothesis that 14-3-3β is pivotal in VSMC survival, we determined whether 14-3-3β overexpression rescued cells from H_2_O_2_-induced caspase 3 activation. 14-3-3β transfection reduced cleaved caspase 3 to the basal level whereas 14-3-3θ and ε transfection did not (Figure 4A). The role of 14-3-3β in controlling apoptosis was investigated by RNA interference. Silencing of 14-3-3β protein expression by 14-3-3β siRNA was accompanied by pronounced caspase 3 at basal cellular state (Figure S4) and abrogated the protective effect of cPGI_2_ (Figure 4B). These results indicate that 14-3-3β upregulation mediates the anti-apoptotic action of PGI_2_ and PPARα agonists. 14-3-3 proteins are capable of binding and sequestering phosphorylated Bad and thus protecting against Bad-triggered apoptosis [23]. To provide evidence for preventing Bad translocation to mitochondria in PGI_2_-treated cells, we isolated mitochondria and analyzed Bad content by Western blotting using HSP60 as a marker. H_2_O_2_-induced Bad translocation to mitochondria was attenuated by cPGI_2_ or Wy14,643 (Figure 4C). In keeping with reduced Bad translocation, Bad binding to 14-3-3β was increased by both cPGI_2_ and Wy14,643 (Figure 4D). These results indicate that through 14-3-3β upregulation, PGI_2_ and PPARα agonists prevent Bad translocation to mitochondria to initiate apoptosis.

### PGI_2_ protects against H_2_O_2_-induced degradation of contractile proteins via 14-3-3β and θ

**Figure 5:**
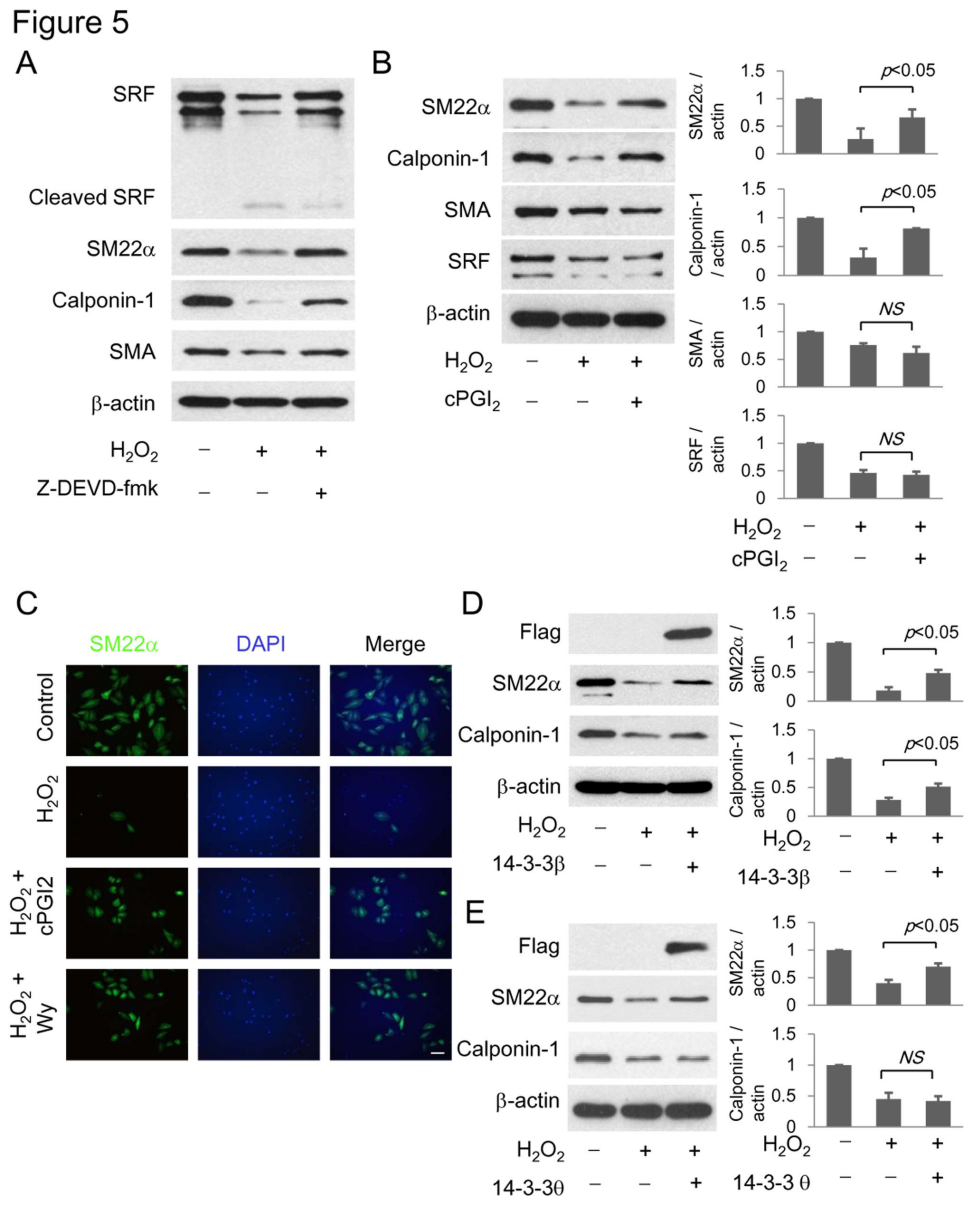
H_2_O_2_ degrades VSMC contractile proteins and SRF via caspase 3. (**A**) Cells were pretreated with Z-DEVD-fmk (20 µM) followed by H_2_O_2_. SRF and contractile proteins were analyzed by Western blotting. (**B**) Cells were pretreated with cPGI_2_ (100 µM) followed by H_2_O_2_. SRF and contractile proteins were analyzed by Western blotting. (**C**) Immunofluorescent staining of SM22α and nuclear staining with DAPI in H_2_O_2_-treated cells in the absence and the presence of cPGI_2_ or Wy14,643. Scale bar = 100 µm. (**D**) VSMCs were transfected with Flag-tagged 14-3-3β or θ vectors. Following H_2_O_2_ treatment, cells were lysed and SM22α and calponin-1 were determined by Western blotting. Each error bar denotes mean±SEM and all blots are representative of n≥3.

Recent studies have provided evidence that VSMC apoptosis triggers VSMCs to acquire proinflammatory and synthetic phenotype [24,25]. It is unclear whether apoptosis influences contractile phenotype. To assess this, we analyzed SM22α, CPN and SMA, in H_2_O_2_-treated cells by Western blotting. All three SM-specific contractile proteins were reduced by H_2_O_2_ (Figure 5A). Serum response factor (SRF) which is considered to be a master regulator of VSMC contractile protein transcription [26,27] was also reduced in H_2_O_2_-treated cells with detectable cleaved SRF fragment (Figure 5A). Pretreatment with caspase 3 inhibitor Z-DEVD-fmk prevented degradation of SM22α, CPN and SMA as well as SRF (Figure 5A). However, PGI_2_ preserved SM22α and CPN but had no significant effect on preserving SMA or SRF (Figure 5B). We next analyzed SM22α by immunofluorescent microscopy. H_2_O_2_ treatment greatly reduced SM22α^+^ cells (Figure 5C). SM22α was detected only in a few intact cells. cPGI_2_ and Wy14,643 increased intact cells accompanied by a higher number of SM22α^+^ cells (Figure 5C). As cPGI_2_ and Wy14,643 upregulated 14-3-3β (Figures 2C and 3B) and 14-3-3β overexpression prevented H_2_O_2_-induced VSMC apoptosis (Figure 4A), we determined whether 14-3-3β prevents H_2_O_2_-induced SM22α and/or CPN degradation. Overexpression of 14-3-3β partially but significantly prevented SM22α and CPN degradation (Figure 5D). For comparison, we evaluated the effect of 14-3-3θ on SM22α and CPN. 14-3-3θ overexpression attenuated H_2_O_2_-induced SM22α degradation but had no effect on CPN degradation (Figure 5E). These results indicate that H_2_O_2_-induced VSMC apoptosis is accompanied by degradation of SRF and contractile proteins. cPGI_2_ partially rescues SM22α and CPN from H_2_O_2_-induced degradation, possibly by 14-3-3β and θ upregulation.

### cPGI_2_ reverses growth factors-induced contractile protein depression via 14-3-3θ

**Figure 6:**
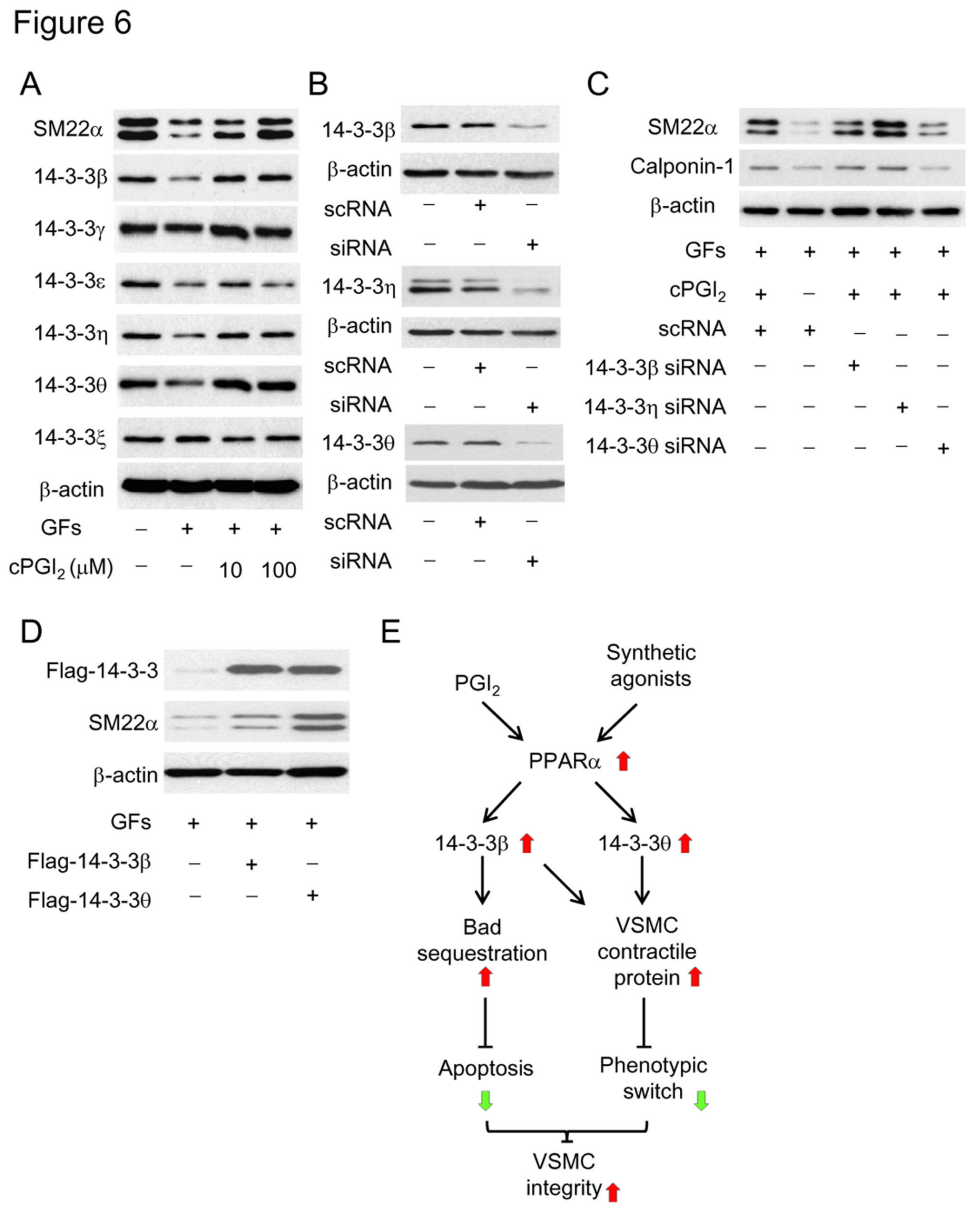
Prostacyclin prevents contractile protein reduction induced by combined growth factors (GFs). (**A**) Cells were pretreated with cPGI_2_ followed by GFs. (**B**) VSMCs were transfected with 14-3-3β, η or θ siRNA or a control scRNA and the respective 14-3-3 proteins were analyzed. (**C**) VSMCs transfected with siRNA of 14-3-3β, η or θ were treated with cPGI_2_ (100 µM) and GFs. SM22α and Calponin-1 in cell lysates were analyzed by Western blotting. (**D**) VSMCs were transfected with Flag-tagged 14-3-3β or θ vectors. Following GFs treatment, cells were lysed and SM22α was determined by Western blotting. All blots are representative of n≥3. (**E**) A schematic illustration of the role of PGI_2_/PPARα/14-3-3β and θ in controlling VSMC apoptosis and contractile phenotype.

In order to gain insights into the control of SM22α and CPN by cPGI_2_, we evaluated the effect of cPGI_2_ on VSMC phenotypic switch induced by multiple growth factors (GFs) including PDGF, FGF2 and EGF. Treatment of A-10 cells with GFs for 48h resulted in reduction of SM22α (Figure 6A) as previously reported [28]. Concurrent analysis of 14-3-3 isoforms shows reduction of 14-3-3β, θ, η and ε by GFs treatment (Figure 6A). cPGI_2_ reversed SM22α decline accompanied by reversal of 14-3-3β, θ, and η. To determine the role of those 14-3-3 isoforms in preventing GFs-induced SM22α decline, we evaluated the effect of individual siRNA on SM22α and CPN protein levels. Figure 6B shows that each siRNA effectively inhibited the expression of 14-3-3 isoforms. 14-3-3θ siRNA abrogated the protective effect of cPGI_2_ on SM22α and CPN while a control scRNA did not (Figure 6C). Neither 14-3-3β siRNA nor 14-3-3η siRNA disrupted the protective effect of cPGI_2_ and paradoxically 14-3-3η siRNA increased SM22α expression (Figure 6C), We next evaluated the influence of 14-3-3θ or β overexpression on SM22α. 14-3-3θ transfection rescued GF-induced depression of SM22α (Figure 6D) while 14-3-3β transfection slightly increased SM22α protein levels (Figure 6D). These results suggest that 14-3-3θ is pivotal in maintaining SM22α in VSMC. As H_2_O_2_-induced apoptosis influences VSMC SM22α and CPN expression, we determined whether GFs have an effect on apoptosis. Combined GFs did not induce VSMC apoptosis nor did they enhance H_2_O_2_-induced caspase 3 cleavage (Figure S5). They attenuated H_2_O_2_-induced PARP and procaspase 3 cleavage (compare Figure S5 with Figure S1).

## Discussion

Our findings provide strong evidence for a crucial role of PPARα in mediating the protective effect of PGI_2_ on VSMCs. As the anti-apoptotic action of PGI_2_ is abrogated by a PPARα antagonist but not a PPARδ antagonist, PPARδ activation by PGI_2_ does not appear to be involved in VSMC protection. PGI_2_ is known to induce VSMC relaxation via membrane I-type prostaglandin (IP) receptor. It remains to be investigated whether IP is involved in the anti-apoptotic action of PGI_2_. As we have previously observed that PGI_2_ protects vascular endothelial cells from H_2_O_2_-induced apoptosis via PPARδ-mediated 14-3-3ε upregulation [13], we determined whether the PGI_2_ protects VSMCs via a similar pathway. The results indicate that although PPAR->14-3-3 pathway is involved, there is a striking difference in the PPAR and 14-3-3 isoforms. In contrast to PPARδ-mediated 14-3-3ε upregulation in VECs, PGI_2_ protects VSMCs from apoptosis via PPARα-mediated 14-3-3β upregulation. Despite the upregulation of the basal level of several 14-3-3 isoforms by PPARα activation or PPARα overexpression, PGI_2_ or Wy14,643 rescue 14-3-3β and θ from H_2_O_2_-induced degradation and only 14-3-3β overexpression is effective in suppressing H_2_O_2_-induced apoptosis. Furthermore, the anti-apoptotic effect of PGI_2_ is abrogated by 14-3-3β siRNA, suggesting the critical role of PPARα-induced 14-3-3β upregulation in mediating the protective effect of PGI_2_. PGI_2_ was previously reported to upregulate 14-3-3ε in VECs which is pivotal in protecting endothelial cell survival [13]. By contrast, PGI_2_ does not upregulate the basal expression of 14-3-3ε nor does it influence 14-3-3ε in H_2_O_2_-treated VSMCs. 14-3-3ε does not appear to play a significant role in protecting VSMC survival as silencing of 14-3-3ε expression with siRNA does not alter H_2_O_2_-induced caspase 3. It is unclear why 14-3-3ε expression responds to PGI_2_ differently in VSMCs vs VECs. Nor is it known why 14-3-3ε protects VEC but not VSMC survival. Further studies are needed to resolve this perplexing issue.

Our data support a link between VSMC apoptosis and changes in contractile proteins. We show that H_2_O_2_-induced apoptosis suppresses the expression of SM22α, CPN and SMA through caspase 3-induced SRF, which is required for transcription of VSMC contractile proteins [29]. Importantly, our results indicate that PGI_2_ is effective in partially preserving SM22α and CPN. Since PGI_2_ does not prevent SRF degradation, we reasoned that PGI_2_ protects SM22α and CPN by a mechanism independent of apoptosis. This notion is supported by the ability of PGI_2_ to protect SM22α against GFs-induced depression. Since GFs do not induce VSMC apoptosis, the protective action of PGI_2_ is independent of counteracting apoptosis. The mechanism by which PGI_2_ preserves SM22α and CPN is unclear but may involve the upregulation of 14-3-3β and θ by PGI_2_. Our results suggest that 14-3-3β and θ may possess distinct functions but act in concerts to protect against apoptosis and preserve SM22α and CPN in VSMCs damaged by oxidative stress and mitogenic stimulation.

Our findings provide novel information about the control of VSMC apoptosis and phenotypic switch by PGI_2_. PGI_2_ is produced by VECs and VSMCs at basal state. Its production by VECs is enhanced by proinflammatory mediators and mechanical stresses. The stress-coupled PGI_2_ production is considered to play a pivotal role in controlling vascular relaxation and platelet reactivity. Our data indicate that PGI_2_ defends against oxidant-induced VSMC apoptosis. Together with our previous report that PGI_2_ protects VECs from H_2_O_2_-induced apoptosis, it may be concluded that PGI_2_ protects vascular integrity when blood vessels are under oxidative and proinflammatory stresses. Our results further show that PGI_2_ controls VSMC phenotypic switch by maintaining SM22α and CPN. At resting state, VSMCs reside in the media layer and assume a contractile phenotype to ensure proper vascular contractility. When the vascular endothelium is injured, VSMCs become highly mobile and assume a synthetic phenotype [30–32], which is considered to play a key role in vascular lesion formation and atherosclerosis [33]. Our results indicate that PGI_2_ is an effective defender against VSMC phenotypic switch by preserving the level of SM22α which is considered to play a key role in maintaining the contractile phenotype [34]. It is interesting that PGI_2_ protects against apoptosis and phenotypic switch via a common PPARα to 14-3-3 signaling pathway. Our results lead us to propose a model of actions as illustrated in Figure 6E. PGI_2_ activates PPARα thereby upregulating 14-3-3β and 14-3-3θ expressions. 14-3-3β binds and sequesters Bad in the cytosol which attenuates caspase 3 activation and apoptosis via the mitochondrial pathway. Caspase 3 degrades SRF and down-regulates the expression of SM22α and other contractile proteins. 14-3-3θ upregulation compensates for the loss of SM22α by stimulating SM22α expression. 14-3-3β overexpression supports SM22α by attenuating Bad-induced caspase 3 activation. Thus, 14-3-3β and 14-3-3θ upregulation work cooperatively to reduce VSMC apoptosis and maintain contractile phenotype. Severe endothelial damage causes deficiency in PGI_2_ production resulting in loss of defense and consequently VSMC apoptosis and VSMC-mediated inflammation, proliferation and intimal hyperplasia. The PGI_2_-PPARα-14-3-3β/θ pathway is thus physiologically important and a therapeutic target for enhancing vascular integrity and preventing vascular diseases.

## Supporting Information

Figure S1 H_2_O_2_ induced PARP and procaspase 3 cleavage in A-10 cells in a concentration-dependent manner.
(PDF)

Figure S2 cPGI_2_ and PPARα agonist prevented A-10 apoptosis induced by serum deprivation for 48 h. PARP and cleaved PARP were analyzed by Western blotting.
(PDF)

Figure S3 Bcl inhibitor, ABT-737, at 1µM did not block cPGI2 protection of H_2_O_2_-induced caspase 3 cleavage.
(PDF)

Figure S4 Suppression of 14-3-3β protein expression with siRNA was accompanied by increased caspase 3 activation.
(PDF)

Figure S5 Combined growth factors (GFs) did not induce apoptosis but attenuated H_2_O_2_-induced PARP and caspase 3 cleavage.
(PDF)
