# Supplementary figures and images for: Prostacyclin and PPARα Agonists Control Vascular Smooth Muscle Cell Apoptosis and Phenotypic Switch through Distinct 14-3-3 Isoforms
Source: PLoS One. 2013 Jul 3;8(7):e69702. doi: 10.1371/journal.pone.0069702 (PMC3701049; doi:10.1371/journal.pone.0069702)

Figure S1

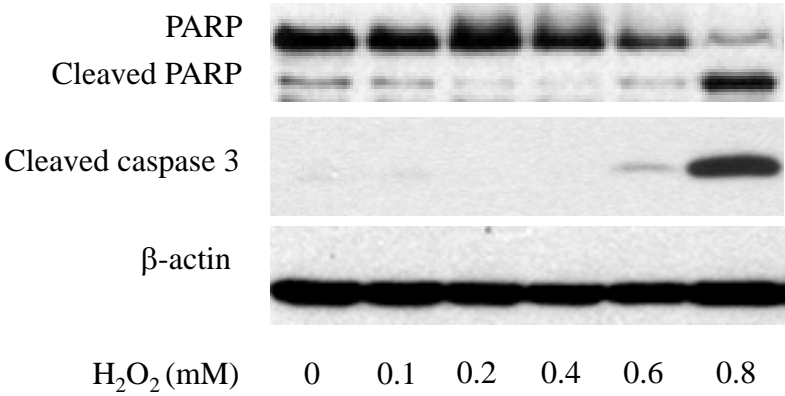

Supplement: Figure S1 — H2O2 induced PARP and procaspase 3 cleavage in A-10 cells in a concentration-dependent manner. (PDF) [file pone.0069702.s001.pdf]

Figure S2

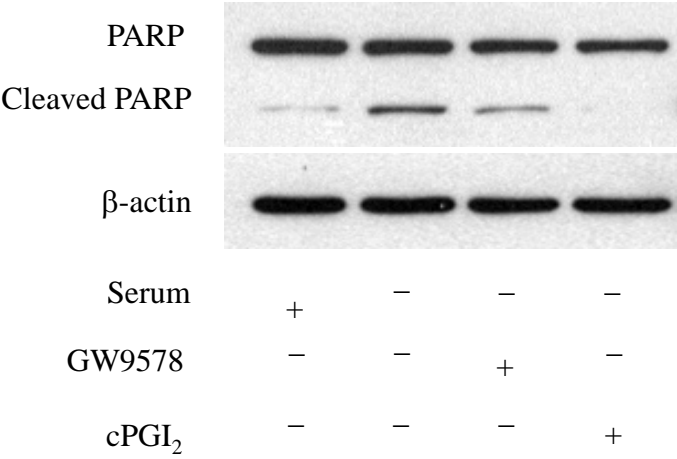

Supplement: Figure S2 — cPGI2 and PPARα agonist prevented A-10 apoptosis induced by serum deprivation for 48 h. PARP and cleaved PARP were analyzed by Western blotting. (PDF) [file pone.0069702.s002.pdf]

Figure S3

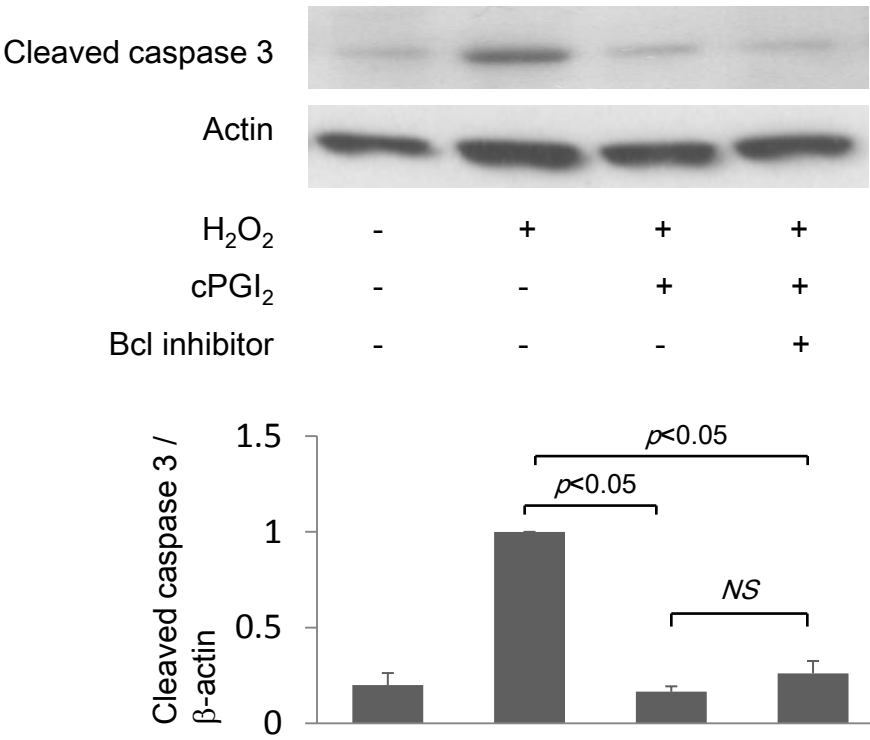

Supplement: Figure S3 — Bcl inhibitor, ABT-737, at 1µM did not block cPGI2 protection of H2O2-induced caspase 3 cleavage. (PDF) [file pone.0069702.s003.pdf]

Figure S4

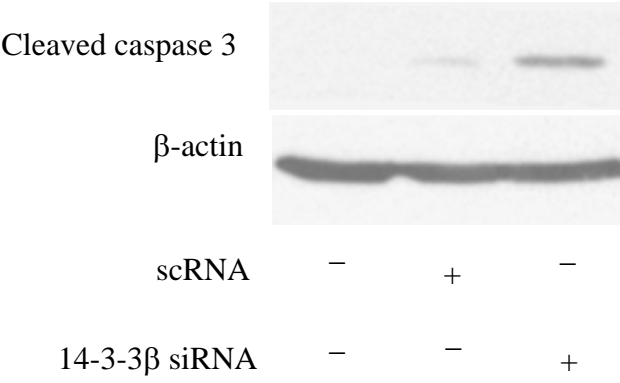

Supplement: Figure S4 — Suppression of 14-3-3β protein expression with siRNA was accompanied by increased caspase 3 activation. (PDF) [file pone.0069702.s004.pdf]

Figure S5

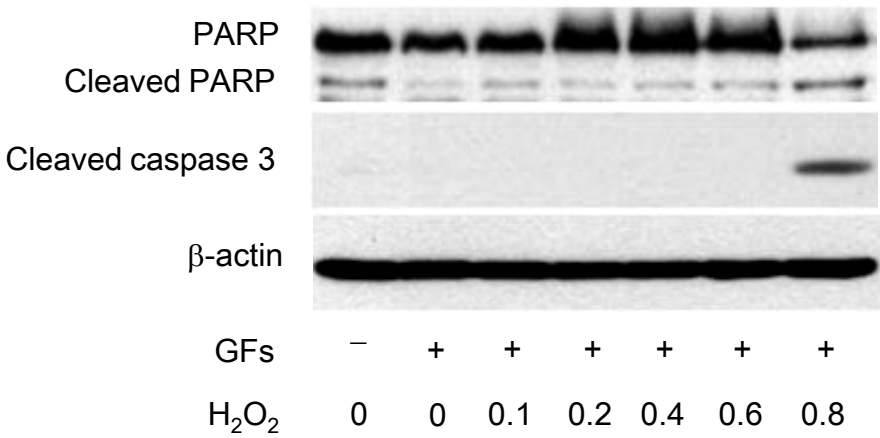

Supplement: Figure S5 — Combined growth factors (GFs) did not induce apoptosis but attenuated H2O2-induced PARP and caspase 3 cleavage. (PDF) [file pone.0069702.s005.pdf]
